# Elevated Type II Secretory Phospholipase A2 Increases the Risk of Early Atherosclerosis in Patients with Newly Diagnosed Metabolic Syndrome

**DOI:** 10.1038/srep34929

**Published:** 2016-12-12

**Authors:** Chang-Qing Sun, Chun-Yan Zhong, Wei-Wei Sun, Hua Xiao, Ping Zhu, Yi-Zhang Lin, Chen-Liang Zhang, Hao Gao, Zhi-Yuan Song

**Affiliations:** 1 Department of Cardiology, Southwest Hospital, The Third Military Medical University, Chongqing, 400038, China; 2 Department of Geriatrics, The First Affiliated Hospital of Xiamen University, Xiamen, 361003, China

## Abstract

A critical association between type II secretory phospholipase A2 (sPLA2-IIa) and established atherosclerotic cardiovascular disease has been demonstrated. However, the contribution of sPLA2-IIa to early atherosclerosis remains unknown. This study investigated the association between early-stage atherosclerosis and sPLA2-IIa in metabolic syndrome (MetS) patients. One hundred and thirty-six MetS patients and 120 age- and gender-matched subjects without MetS were included. Serum sPLA2-IIa protein levels and activity were measured using commercial kits. Circulating endothelial activation molecules (vascular cell adhesion molecule-1 (VCAM-1), intercellular adhesion molecule-1 (ICAM-1), E-selectin, and P-selectin), and carotid intima-media thickness (cIMT), were measured as parameters of vascular endothelial dysfunction and early atherosclerosis. MetS patients exhibited significantly higher sPLA2-IIa protein and activity levels than the controls. Both correlated positively with fasting blood glucose and waist circumference in MetS patients. Additionally, MetS patients exhibited strikingly higher levels of endothelial activation molecules and increased cIMT than controls. These levels correlated positively with serum sPLA2-IIa protein levels and activity. Moreover, multivariate analysis showed that high sPLA2-IIa protein and activity levels were independent risk factors of early atherosclerosis in MetS patients. This study demonstrates an independent association between early-stage atherosclerosis and increased levels of sPLA2-IIa, implying that increased sPLA2-IIa may predict early-stage atherosclerosis in MetS patients.

The metabolic syndrome (MetS) is defined as an aggregation of risk factors that include central obesity, atherogenic dyslipidemia, elevated plasma glucose, and elevated blood pressure^1^. Patients with these features commonly manifest pro-inflammatory and pro-thrombotic states that appear to directly promote the onset and progression of atherosclerotic cardiovascular disease^2^. Inflammation is thought to play a pivotal role in the pathogenesis of atherosclerosis and to trigger subsequent thrombotic complications^3^. Circulating levels of inflammatory biomarkers are increased in patients with established^4^ and progressing^5^,^6^ coronary artery disease (CAD). An analysis suggests that 6–7% of all-cause mortality and 12–17% of cardiovascular diseases are attributable to the MetS^7^. Furthermore, coronary heart disease, cardiovascular disease, and total mortality are significantly higher in patients with the MetS than in those without^8^. Therefore, early detection of subclinical atherosclerosis in MetS patients is essential to reduce the risk of morbidity and mortality from cardiovascular complications related to this disorder.

Secretory phospholipase A2 (sPLA2) enzymes hydrolyze the sn-2 ester bond in glyceroacyl phospholipids of lipoproteins and cell membranes, producing non-esterified fatty acids and lysophospholipids^9^. Group IIa sPLA2 (sPLA2-IIa), a well-studied member of the sPLA2 family, was first isolated and purified from rheumatoid arthritis fluids. sPLA2-IIa is a low-molecular-weight (14 kDa) Ca^2+^-dependent enzyme, expressed in macrophages, platelets, vascular smooth muscle cells and atherosclerotic lesions^10^. Expression of sPLA2-IIa is up-regulated in response to cytokines such as interferon-γ (IFN-γ), tumour necrosis factor-α (TNF-α), interleukin-1β (IL-1β) and oxidized low-density lipoprotein (LDL)^10^,^11^. In healthy individuals, serum sPLA2 activity provides prognostic value in predicting incident CAD^12^. In subjects with low-to-normal LDL levels and no known cardiovascular disease, sPLA2-IIa is a measurable biomarker to assess the prognostic impact of inflammation on the risk of CAD^13^. In CAD patients, an increase in circulating sPLA2-IIa levels is a significant risk factor of clinical coronary events during follow-up^14^,^15^,^16^. Although numerous studies have focused on the relationship between sPLA2-IIa and established atherosclerotic cardiovascular disease, the contribution of this enzyme to the early-stage atherosclerosis of MetS patients remains unknown.

Previous studies indicated that circulating endothelial activation molecules (i.e. vascular cell adhesion molecule-1 (VCAM-1), intercellular adhesion molecule-1 (ICAM-1), E-selectin and P-selectin)^17^,^18^, and carotid intima-media thickness (cIMT)^19^ are indicators of vascular endothelial dysfunction and early-stage atherosclerosis. The present study investigated the association between sPLA2-IIa protein levels and activity, and early-stage atherosclerosis, in MetS patients.

## Results

### Characteristics of the study population

One hundred and thirty-six MetS patients and 120 age- and gender-matched subjects without MetS were included in this study. The controls and MetS patients were similar in age (*p* = 0.441), gender (*p* = 0.657), educational level (*p* = 0.673), total cholesterol (TC) (*p* = 0.406), and smoking history (*p* = 0.808). Compared to control subjects, MetS patients exhibited higher waist circumference (WC), systolic blood pressure (SBP), diastolic blood pressure (DBP), triglyceride (TG), and fasting blood glucose (FBG), and lower levels of high-density lipoprotein cholesterol (HDL-C) (all *p* < 0.001) (Table 1).

### Serum levels of sPLA2-IIa protein, sPLA2 activity, and endothelial activation molecules and cIMT

Compared with control subjects, serum sPLA2-IIa protein and activity levels were significantly higher in MetS patients (sPLA2-IIa protein: *p* = 0.008; sPLA2 activity: *p* = 0.002). There were also striking differences in serum ICAM-1, VCAM-1, E-selectin, and P-selectin in MetS patients (ICAM-1: *p* = 0.002; VCAM-1: *p* < 0.001; E-selectin: *p* = 0.020; P-selectin: *p* = 0.004). In addition, MetS patients had thicker cIMT than controls (*p* = 0.013) (Table 2). These data suggested that systematic inflammation had occurred in patients with MetS, which accompanied with vascular endothelial dysfunction and early atherosclerosis.

### Correlations between serum sPLA2-IIa protein and activity levels, and metabolic components in MetS subjects

We used partial correlation analyses to investigate the correlations between serum sPLA2-IIa protein levels and activity, and metabolic components with adjustments for age, gender, educational level, and smoking history. In the model that included all participants, serum sPLA2-IIa protein and activity levels correlated positively with WC and FBG (Table 3). In the model limited to MetS patients, serum sPLA2-IIa protein and activity levels remained positively correlated with WC (*r* = 0.254, *p* = 0.0311; *r* = 0.290, *p* = 0.015) and FBG (*r* = 0.276, *p* = 0.024; *r* = 0.241, *p* = 0.043).

### Correlations between serum sPLA2-IIa levels, endothelial activation molecules, and cIMT in MetS patients

**Figure 1:**
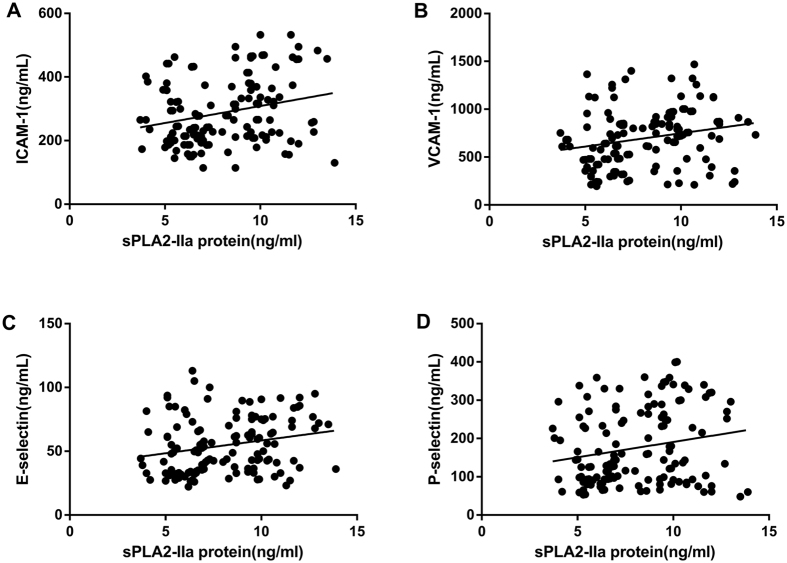
Correlations of serum sPLA2-IIa protein levels with the levels of circulating endothelial activation molecules in patients with MetS. Positive correlations were found between serum sPLA2-IIa protein levels and serum (**A**) ICAM-1 (r = 0.308, *p* = 0.011), (**B**) VCAM-1 (r = 0.430, *p* < 0.001), (**C**) E-selectin (r = 0.374, *p* = 0.002), and (**D**) P-selectin (r = 0.259, *p* = 0.033). Spearman correlation analyses.

**Figure 2:**
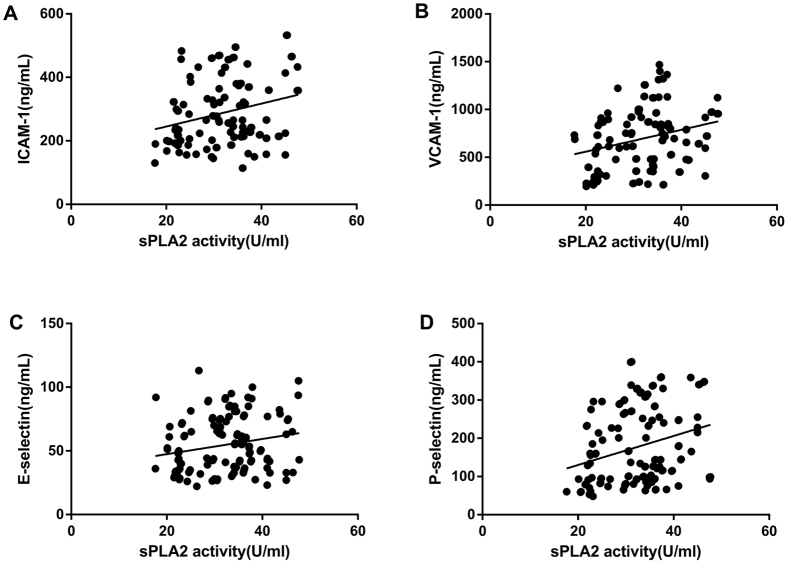
Correlations of serum sPLA2 activity with the levels of circulating endothelial activation molecules in patients with MetS. Positive correlations were found between serum sPLA2 activity levels and the levels of serum (**A**) ICAM-1 (r = 0.282, *p* = 0.020), (**B**) VCAM-1 (r = 0.288, *p* = 0.017), (**C**) E-selectin (r = 0.337, *p* = 0.005), and (**D**) P-selectin (r = 0.403, *p* < 0.001). Spearman correlation analyses.

**Figure 3:**
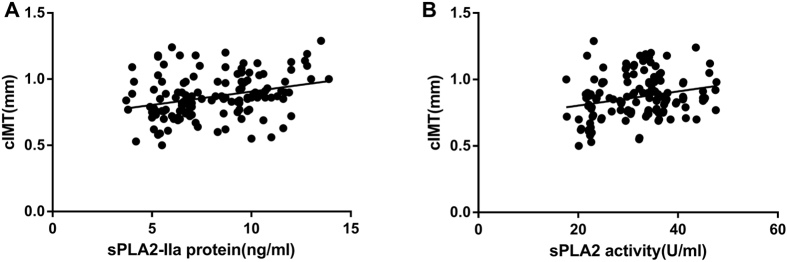
Correlations of serum sPLA2-IIa protein and activity levels with cIMT in patients with MetS. Both sPLA2-IIa (**A**) protein (r = 0.348, *p* = 0.004) and (**B**) activity (r = 0.255, *p* = 0.036) levels correlated positively with cIMT in MetS patients. Spearman correlation analyses.

Spearman’s correlation analysis was used to determine the linear correlation between sPLA2-IIa protein and activity levels, endothelial activation molecules, and cIMT in patients with MetS (Figs 1, 2 and 3). In MetS patients, serum sPLA2-IIa protein levels correlated positively with serum ICAM-1 (*r* = 0.308, *p* = 0.011), VCAM-1 (*r* = 0.430, *p* < 0.001), E-selectin (*r* = 0.374, *p* = 0.002), and P-selectin (*r* = 0.259, *p* = 0.033) (Fig. 1). Serum sPLA2 activity levels also correlated positively with serum ICAM-1 (*r* = 0.282, *p* = 0.020), VCAM-1 (*r* = 0.288, *p* = 0.017), E-selectin (*r* = 0.337, *p* = 0.005), and P-selectin (*r* = 0.403, *p* < 0.001) (Fig. 2). Additionally, sPLA2-IIa protein (*r* = 0.348, *p* = 0.004) and activity (*r* = 0.255, *p* = 0.036) levels were both positively correlated with cIMT (Fig. 3). In order to avoid potential confounding factors, we used partial correlation analyses to investigate the correlations between sPLA2-IIa protein and activity levels, and endothelial activation molecules. Adjustments were made for age, gender, educational level, and smoking history. After these adjustments, serum sPLA2-IIa protein levels exhibited significant associations with ICAM-1 (*r* = 0.289, *p* = 0.014), VCAM-1 (*r* = 0.270, *p* = 0.032), E-selectin (*r* = 0.324, *p* = 0.007), and P-selectin (*r* = 0.248, *p* = 0.040) in MetS patients. Additionally, serum sPLA2 activity showed significant associations with ICAM-1 (*r* = 0.269, *p* = 0.034), VCAM-1 (*r* = 0.274, *p* = 0.031), E-selectin (*r* = 0.316, *p* = 0.009), and P-selectin (*r* = 0.279, *p* = 0.028) in MetS patients. When we applied the Benjamini-Hochberg method to correct for multiple testing (FDR < 0.05), these correlations remained significant. In subjects without MetS, the serum sPLA2-IIa protein and activity levels did not correlated with endothelial activation molecules and cIMT by partial correlation analyses (Supplementary Table S1).

### Logistic Regression Analysis for Predictors of Early Atherosclerosis in MetS Patients

The effect of sPLA2-IIa protein and sPLA2 activity on incident high cIMT was tested respectively by entering ordinal terms for metabolic variables and endothelial activation molecules in the logistic regression models. The variables associated with cIMT at univariate analysis entered into the unconditional logistic models including: age, gender, SBP, TG, WC, FBG, and endothelial activation molecules (VCAM-1, E-selectin, ICAM-1 and P-selectin) (Supplementary Table S2). Outcome variables (men: cIMT ≥ 0.96 mm; women; cIMT ≥ 0.85 mm) were employed to assess whether sPLA2-IIa protein and activity levels enhanced thickening of carotid vessel wall (Table 4). Increased sPLA2-IIa protein and activity levels were associated with increased risks of enhanced cIMT (OR = 1.176, 95% CI: 1.118–1.235, *p* = 0.003; OR = 1.142, 95% CI: 1.098–1.178, *p* = 0.004) after adjusted for age and gender. These relations remained significant after adjustment for metabolic variables (SBP, TG, WC, and FBG) (OR = 1.140, 95% CI: 1.030–1.155, *p* = 0.010; OR = 1.086, 95% CI: 1.035–1.161, *p* = 0.013). When both metabolic variables and endothelial activation molecules were included in the logistic regression models, the effects of sPLA2-IIa protein and sPLA2 activity on incident high cIMT were attenuated but remained clinically and statistically significant (OR = 1.113, 95% CI: 1.019–1.148, *p* = 0.022; OR = 1.074, 95% CI: 1.012–1.150, *p* = 0.038).

## Discussion

Our study demonstrated that patients with MetS that were free of cardiovascular disease had elevated serum sPLA2-IIa protein and activity values. In MetS patients, serum sPLA2-IIa protein and activity levels were associated independently with markers of endothelial activation. Moreover, both sPLA2-IIa protein and activity levels associated with increased cIMT in multivariable analysis, the association between levels of sPLA2-IIa and incident high cIMT remained significant after sequentially controlling for MetS components and endothelial activation molecules. Our findings provide novel insights by demonstrating that elevated sPLA2-IIa contributes to the increased risk of developing early-stage atherosclerosis and may contribute to its atherogenicity in patients with MetS.

sPLA2-IIa is a key enzyme in metabolising lipoproteins and modifying atherogenic LDL^20^. Therefore, sPLA2-IIa may be involved in metabolism-related disorders. In the current study, levels of both serum sPLA2-IIa protein and activity were increased significantly in MetS patients. Importantly, sPLA2-IIa protein and activity levels were significantly and positively associated with the MetS components WC and FBG. Furthermore, these correlations remained after adjusting for the confounders of age, gender, and smoking history.

In a previous study, we found that visceral adipose tissue was an important source of sPLA2-IIa, both in atherosclerotic and normal rats^21^. Dutour *et al*.^22^ also reported an increased expression of sPLA2-IIa in adipose tissue of CAD patients. These studies support the perspective that increased sPLA2-IIa levels in MetS patients may partly result from the upregulation of its production in adipose tissue. Previous observations have linked sPLA2-IIa with insulin resistance^23^ and type 2 diabetes^24^. In addition, *in vitro* experiments demonstrated that high concentrations of glucose had a significant stimulatory effect on sPLA2-IIa expression by enhancing the activity of the rat sPLA2-IIa-promoter^25^. This suggests that elevated serum sPLA2-IIa protein and activity maybe explained partly by increased FBG in MetS patients.

Recruitment and adhesion of monocytes to the arterial endothelial lining is one of the earliest detectable events during atherogenesis^26^. Endothelial activation molecules (i.e. E-selectin, P-selectin, ICAM-1, and VCAM-1) are considered to play crucial roles in the cascade of cell interactions that mediate extravasation and migration of inflammatory cells into the vascular endothelium^27^. Therefore, these cytokines are regarded as surrogate markers of low-grade vascular inflammation, reflecting endothelial dysfunction. Our study suggested that all endothelial activation molecules were positively associated with cIMT of MetS patients. When endothelial activation molecules were included in the multivariable analysis models, the OR of incident high cIMT associated with sPLA2-IIa protein and sPLA2 activity was attenuated to 3.1% and 4.9% respectively. The association of endothelial activation molecules with cIMT and their effect on incident high cIMT reflected the occurrence of vascular endothelial dysfunction in early atherosclerosis of MetS patients.

In the present study, we demonstrated that endothelial activation molecule levels were significantly higher in MetS patients than controls. Serum sPLA2-IIa protein and activity levels were strongly and independently correlated with endothelial activation molecules in MetS patients. Similar to our study, Leinone *et al*.^23^ reported that sPLA2-IIa levels were associated positively with ICAM-1 and E-selectin, and correlated with insulin resistance and obesity in type 2 diabetic patients. At the cellular level, sPLA2 increased the expression of ICAM-1 and VCAM-1 significantly in human umbilical vein endothelial cells by oxidised LDL^28^. Therefore, the elevated sPLA2-IIa levels observed in our patients may contribute to increasing the levels of endothelial activation molecules, which may then initiate atherosclerosis pathogenesis in MetS patients.

From a structural perspective, cIMT can predict future clinical cardiovascular events because it is an established biomarker of atherosclerosis and subclinical organ damage^29^. Enhanced cIMT is also an established predictor for early impairment of the vascular endothelium^30^,^31^. Based on these findings, we measured cIMT and found that serum sPLA2-IIa protein and activity levels were both independent predictors of early structural changes in the arteries of MetS patients. Similar to our observations, a recent study also reported elevated sPLA2 activity levels in young adults (aged 24 to 39 years) with MetS^32^. However, their analysis suggested that sPLA2 activity does not play a significant role in explaining the increased risk of cIMT associated with MetS. Furthermore, Braamskamp *et al*.^33^ observed that sPLA2-IIa protein and activity levels were not associated significantly with cIMT in children with familial hypercholesterolemia. The apparent discrepancy between our result and these studies may be due to the different ages and complications of the subjects. Overall, more clinical data are required to establish the effect of sPLA2-IIa on early atherogenesis in patients of different ages and with various complications.

Accumulating evidence from basic research studies and clinical trials suggests that sPLA2-IIa plays a central role in inflammatory reactions and the recruitment of inflammatory cells to endothelium in the pathogenesis of atherosclerosis. *In vitro*, sPLA2-IIa induces mononuclear cell differentiation and increases their adhesive and migratory capabilities^34^. Additionally, sPLA2-IIa has been detected in vascular smooth muscle cells from both the medial and intimal layers, and in macrophage-rich regions of atherosclerotic plaques, and may induce foam cell formation and LDL modifications^20^,^35^. These changes may result in an increased retention of foam cells and LDL particles in the arterial wall. In human sPLA2-IIa transgenic mice, macrophage-specific expression of sPLA2-IIa results in accelerated atherogenesis by increasing LDL oxidation^36^. Furthermore, sPLA2 mediates the LDL-elicited release of arachidonic acid, a known precursor of various proinflammatory mediators such as leukotrienes and prostaglandins^37^. Nevertheless, the causal relationship between early atherosclerosis and sPLA2-IIa levels remains to be clarified by further investigations.

Because the present study was observational, we cannot prove any causal relationship between early atherosclerosis and sPLA2-IIa. We used mean cIMT as an indicator of early atherosclerosis. However, a previous study suggested that the maximum IMT of the internal carotid artery may be more sensitive to cardiovascular disease^38^. Our study population was relatively small and racially homogenous, so the results can be generalised only to Chinese subjects.

In summary, the results of this study demonstrated that increased sPLA2-IIa protein and activity levels in the serum were strongly associated with the presence of MetS and its components. In addition, sPLA2-IIa levels and activity correlated with markers of endothelial activation and early atherosclerosis in patients with MetS. The presence of elevated sPLA2-IIa protein and activity may identify an especially high risk of early atherosclerosis in MetS patients.

## Methods

### Study population

A case-control study was conducted among participants recruited from our institute from January 2012 to December 2015. A total of 136 newly diagnosed MetS subjects, and 120 age- and gender-matched subjects without MetS, participated in the study. All the participants in this study were Chinese. Subjects were excluded for the following reasons: (1) established cardiovascular diseases, defined as a history of CAD, cerebral artery disease, or peripheral artery disease; (2) infectious or immunological disease; and (3) severe pulmonary, hepatic, or renal diseases, or any type of tumour. Additionally, participants were excluded if they were taking any medications known to affect inflammatory conditions including steroids, non-steroidal anti-inflammatory agents, antihyperlipidemic drugs, antihypertensive drugs, hypoglycemic drugs, cyclosporine, and antibiotics. The study was performed according to the Helsinki Declaration and was approved by the Medical Research Ethics Committee of the Southwest Hospital. Informed consent was acquired from each participant before the collection of blood specimens.

### MetS diagnosis and blood sampling

MetS was diagnosed based on a modified combination of the National Cholesterol Education Program-Adult Treatment Panel III guideline and the Chinese guideline for prevention and treatment of dyslipidemia in adults^39^. Diagnosis required at least three of the following components: [1] WC ≥ 90 cm in males or 85 cm in females; [2] TG ≥ 150 mg/dL; [3] high density lipoprotein cholesterol (HDL-C) less than 40 mg/dL; [4] blood pressure ≥ 130/85 mmHg; and [5] FBG ≥ 100 mg/dL. Medical histories including age, gender, height, weight, and current medications were collected upon admission. After a 12-h fasting period, venous blood samples were collected in either EDTA-treated or plain tubes between 06:00 and 07:00, then centrifuged immediately to yield either plasma or serum, and stored at −80 °C until use.

### Blood analyses

TC, HDL-C, TG, and Fasting plasma glucose concentration (FPG) were measured by the autoanalyser (HITACHI, Tokyo, Japan) with commercially available kits (Randox Laboratories, Crumlin, UK). Serum sPLA2-IIa protein was determined using a sandwich-type enzyme-linked immunosorbent assay (ELISA) kit (Cayman Chemicals, Ann Arbor, MI, USA). Serum sPLA2 activity was measured by a selective fluorometric assay (Cayman Chemicals) by using diheptanoyl thio-phosphocholine as fluorescent substrate. We used a solution of 0.1U bee venom PLA2 as a positive control to hydrolyze fluorescent substrate completely. The hydrolysis of substrate in the absence of serum sample was used as negative control. The intra-assay coefficient of variation was 4.2%. ELISAs were used to determine VCAM-1, ICAM-1, E-selectin, and P-selectin levels (R & D Systems, Minneapolis, MNUSA). All measurements were performed according to the manufacturers’ instructions. Each sample was tested in duplicate, and the means of the duplicates were used for the statistical analysis.

### Carotid intima-media thickness

Measurements of cIMT were performed on an Acuson Sequioa 512 ultrasound machine (7.0 MHz probe) (Siemens, Malvern, PA, USA) by one blinded sonographer. Images of the right and left common carotid arteries, the carotid bulb, and the internal carotid far wall segments were acquired. Analyses were done off-line with image-analysis software by another blinded sonographer. Mean cIMT was defined as the mean IMT of the right and left common carotid arteries, the carotid bulb, and the internal carotid far wall segments^40^.

### Statistical analyses

Statistical analyses were performed with SPSS 18.0 for Windows (SPSS, Chicago, IL, USA). Continuous variables are expressed as means ± standard deviation (SD). Two-tailed independent t-tests and Mann-Whitney U tests were used to compare parameters between the two groups. Frequency was tested with the chi-squared test. Partial correlation analyses were used to investigate the correlations between serum sPLA2-IIa protein and activity levels, and the MetS components. Spearman correlation was used to determine the linear correlation between the serum sPLA2-IIa protein and activity levels and endothelial activation molecules, partial correlation analyses was used to control the confounders. Benjamini-Hochberg correction for multiple testing was done (FDR < 0.05). Because of the skewed distribution, sPLA2-IIa protein, sPLA2 activity, ICAM-1, VCAM-1, E-selectin, and P-selectin were log-transformed to approximate a normal distribution before entering regression analysis. We calculated the Odds Ratio (OR) corresponding 95% confidence intervals (95% CIs) by unconditional logistic regression analysis to select the variables independently associated with the risk of high cIMT (men: cIMT ≥ 0.96 mm; women; cIMT ≥ 0.85 mm) in MetS patients. The variables associated with cIMT at univariate analysis entered into the logistic models. A 2-tailed p value < 0.05 was considered statistically significant.

## Additional Information

**How to cite this article**: Sun, C.-Q. *et al*. Elevated Type II Secretory Phospholipase A2 Increases the Risk of Early Atherosclerosis in Patients with Newly Diagnosed Metabolic Syndrome. *Sci. Rep.* **6**, 34929; doi: 10.1038/srep34929 (2016).

**Publisher's note:** Springer Nature remains neutral with regard to jurisdictional claims in published maps and institutional affiliations.

## Supplementary Material

Supplementary Information

## Figures and Tables

**Table 1:** Characteristics of the study population.

	Controls (n = 120)	MetS (n = 136)	*P* value
Age (years)	54.50 ± 7.68	52.74 ± 8.40	0.441
Gender, male, n (%)	31 (51.76)	36 (52.94)	0.657
Education (years)	15.25 ± 4.65	14.21 ± 2.49	0.673
Current smokers, n (%)	24 (40.00)	28 (41.18)	0.808
WC (cm)	83.46 ± 9.88	91.43 ± 8.39	<0.001
SBP (mmHg)	112.30 ± 13.97	135.49 ± 16.46	<0.001
DBP (mmHg)	71.80 ± 7.71	81.41 ± 9.35	<0.001
TG (mg/dL)	110.18 ± 53.42	212.64 ± 76.76	<0.001
HDL-C (mg/dL)	58.92 ± 0.51	46.96 ± 0.71	<0.001
TC (mg/dL)	199.50 ± 1.69	201.00 ± 1.41	0.406
FBG (mg/dL)	89.39 ± 13.71	108.01 ± 20.13	<0.001

Data are given as means ± SD. Statistical significance was determined by the two-tailed independent t-test or chi-squared test, as appropriate. Abbreviations: WC, waist circumference; SBP, systolic blood pressure; DBP, diastolic blood pressure; TG, triglyceride; HDL-C, high-density lipoprotein cholesterol; TC, total cholesterol; FBG, fasting blood glucose.

**Table 2:** Serum sPLA2-IIa protein and activity levels, endothelial activation molecule levels, and cIMT in controls and MetS patients.

	Controls (n = 120)	MetS (n = 136)	*P* value
sPLA2-IIa protein (ng/mL)	4.69 ± 2.40	7.83 ± 2.58	0.008
sPLA2 activity (U/mL)	22.49 ± 10.37	31.21 ± 7.00	0.002
ICAM-1 (ng/mL)	166.50 ± 82.01	287.12 ± 103.90	0.002
VCAM-1 (ng/mL)	469.61 ± 164.91	700.63 ± 313.80	<0.001
E-selectin (ng/mL)	45.37 ± 18.35	53.20 ± 21.23	0.020
P-selectin (ng/mL)	137.11 ± 71.38	166.16 ± 100.27	0.004
cIMT (mm)	0.61 ± 0.25	0.87 ± 0.17	0.013

Data are given as means ± SD. *P* values for the differences between groups were calculated by the two-tailed independent t-test. Abbreviations: VCAM-1, vascular cell adhesion molecule-1; ICAM-1, intercellular adhesion molecule-1; cIMT, carotid intima-media thickness.

**Table 3:** sPLA2-IIa protein and activity levels in MetS patients.

	sPLA2-IIa protein		sPLA2 activity	
	*r*	*P*	*r*	*P*
WC (cm)	0.184	0.037	0.219	0.017
SBP (mmHg)	0.126	0.158	0.084	0.304
DBP (mmHg)	0.055	0.611	0.110	0.219
TG (mg/dL)	0.136	0.145	0.064	0.480
HDL-C (mg/dL)	−0.074	0.402	0.116	0.208
FBG (mg/dL)	0.190	0.031	0.181	0.042

Partial correlation was used in the analysis.

**Table 4:** OR of Serum sPLA2-IIa protein and sPLA2 activity Levels With cIMT by Multivariate Logistic Regression Analysis (men: cIMT ≥ 0.96 mm; women: cIMT ≥ 0.85 mm).

	sPLA2-IIa protein			sPLA2 activity		
	OR	95% CI	*p*	OR	95% CI	*p*
Model 1	1.176	1.118–1.235	0.003	1.142	1.098–1.178	0.004
Model 2	1.160	1.105–1.206	0.006	1.141	1.098–1.176	0.006
Model 3	1.156	1.097–1.208	0.006	1.137	1.090–1.177	0.007
Model 4	1.152	1.094–1.204	0.009	1.129	1.084–1.169	0.009
Model 5	1.140	1.030–1.155	0.010	1.086	1.035–1.161	0.013
Model 6	1.113	1.019–1.148	0.022	1.074	1.012–1.150	0.038

CI = confidence interval, OR = odds ratio. Model 1: Adjusted for age and gender. Model 2: Adjusted for age, gender, and SBP. Model 3: Adjusted for age, gender, SBP, and TG. Model 4: Adjusted for age, gender, SBP, TG, and WC. Model 5: Adjusted for age, gender, SBP, TG, WC, and FBG. Model 6: Adjusted for age, gender, SBP, TG, WC, FBG, and endothelial activation molecules.
