# Supplementary material for: Elevated Type II Secretory Phospholipase A2 Increases the Risk of Early Atherosclerosis in Patients with Newly Diagnosed Metabolic Syndrome
Source: Sci Rep. 2016 Dec 12;6:34929. doi: 10.1038/srep34929 (PMC5150250; doi:10.1038/srep34929)
Supplement: Supplementary Information [file srep34929-s1.pdf]

Elevated Type II Secretory Phospholipase A2 Increases the Risk of Early Atherosclerosis in Patients with Newly Diagnosed Metabolic Syndrome

Chang-Qing Sun[1, 2], Chun-Yan Zhong[1], Wei-Wei Sun[1], Hua Xiao[1], Ping Zhu[1], Yi-Zhang Lin[1], Chen-Liang Zhang[1], Hao Gao[1], Zhi-Yuan Song[1*]

[1]Department of Cardiology, Southwest Hospital, The Third Military Medical University, Chongqing, 400038, China.

[2]Department of Geriatrics, The First Affiliated Hospital of Xiamen University, Xiamen, 361003, China.

[*]Corresponding. Zhi-Yuan Song (email: zysong2010@126.com)

Supplementary Tables

Supplementary Table S1. The correlations of sPLA2-IIa protein and sPLA2 activity levels with endothelial activation molecules and cIMT in subjects without MetS.

	sPLA2-IIa protein		sPLA2 activity	
	r	*p*	*r*	*p*
ICAM-1	0.120	0.095	0.136	0.056
VCAM-1	0.047	0.576	0.110	0.124
E-selectin	0.125	0.087	0.097	0.191
P-selectin	0.131	0.068	0.105	0.165
cIMT	0.063	0.313	0.089	0.218

Partial correlation analysis, adjusted for age, gender, education, and smoking history.

Abbreviations: VCAM-1, vascular cell adhesion molecule-1; ICAM-1, intercellular adhesion molecule-1; cIMT, carotid intima-media thickness.

Supplementary Table S2. Univariate ORs for high cIMT (men: cIMT ≥ 0.96 mm; women; cIMT ≥ 0.85 mm)

Variable	OR	95% CI	*p*
Age(> 65 years)	3.93	2.68-5.84	0.009
Gender(Male)	1.50	1.20-3.32	0.029
Current smoking(yes)	0.66	0.43-1.21	0.650
SBP(≥ 130 mmHg)	1.32	1.02-1.72	0.031
DBP(≥ 85 mmHg)	0.82	0.83-1.03	0.416
TG(≥ 150 mg/dL)	1.35	1.03-1.72	0.030
HDL-C(< 40 mg/dL)	0.97	0.82-1.16	0.350
WC(≥ 90cm in males, ≥85cm in females)	1.58	1.25-3.37	0.025
FBG(≥ 100 mg/dL)	4.21	2.56-6.85	0.008
ICAM-1	4.71	2.93-7.57	0.002
VCAM-1	3.22	1.57-5.86	0.016
E-selectin	3.14	1.19-5.20	0.017
P-selectin	5.10	3.17-8.45	＜0.001
sPLA2-IIa protein	4.97	2.13-8.07	＜0.001
sPLA2 activity	3.58	1.22-6.12	0.011

Abbreviations: SBP, systolic blood pressure; DBP, diastolic blood pressure; TG, triglyceride; HDL-C, high-density lipoprotein cholesterol; WC, waist circumference; FBG, fasting blood glucose.
